# Enhanced morphological and functional differences of pancreatic cancer with epithelial or mesenchymal characteristics in 3D culture

**DOI:** 10.1038/s41598-019-47416-w

**Published:** 2019-07-26

**Authors:** Yuuki Shichi, Norihiko Sasaki, Masaki Michishita, Fumio Hasegawa, Yoko Matsuda, Tomio Arai, Fujiya Gomi, Junko Aida, Kaiyo Takubo, Masashi Toyoda, Hisashi Yoshimura, Kimimasa Takahashi, Toshiyuki Ishiwata

**Affiliations:** 1 Department of Veterinary Pathology, School of Veterinary Medicine, Nippon Veterinary and Life Science University, Tokyo, 180-8602 Japan; 2 Research Team for Geriatric Medicine (Vascular Medicine), Tokyo Metropolitan Institute of Gerontology, Tokyo, 173-0015 Japan; 3 Division of Aging and Carcinogenesis, Research Team for Geriatric Pathology, Tokyo Metropolitan Institute of Gerontology, Tokyo, 173-0015 Japan; 4 Department of Pathology, Tokyo Metropolitan Geriatric Hospital and Institute of Gerontology, Tokyo, 173-0015 Japan; 5 Division of Physiological Pathology, Department of Applied Science, School of Veterinary Nursing and Technology, Nippon Veterinary and Life Science University, Tokyo, 180-8602 Japan

**Keywords:** Cancer microenvironment, Pancreatic cancer

## Abstract

Pancreatic cancer, composed of heterogeneous cancer cells, alters epithelial to mesenchymal features during growth and metastasis. In this study, we aimed to characterize pancreatic ductal adenocarcinoma (PDAC) cells showing epithelial or mesenchymal features in 3D culture. In 3D culture, PK-1 cells had high *E-cadherin* and low *vimentin* expression and exhibited a round-like appearance encircled by flat cells. PANC-1 cells had high *vimentin* and low *E-cadherin* expression and formed grape-like spheres. PK-1 cells had secretary granules and many microvilli, desmosomes, and adherens junctions, while PANC-1 cells had few microvilli, adherens junction, and no desmosomes. Cytokeratin 7, trypsin, CA19-9, and E-cadherin were highly expressed in PK-1 cells but not in PANC-1 cells. Ki-67 was diffusely expressed in PANC-1 spheres but was restricted to the peripheral flat cells of PK-1 spheres. PANC-1 and PK-1 cells were positive for transforming growth factor (TGF) β receptor II and phosphorylated smad2/3, but PK-1 cells were smad4 negative. Taken together, 3D culture enhanced morphofunctional differences of PDAC cells showing epithelial or mesenchymal characteristics, and epithelial phenotype maintenance may be due to the ineffectiveness of the TGF- β pathway. Clarification of heterogeneity using 3D culture may be useful for development of individualized diagnostic and therapeutic methods in patients with PDAC.

## Introduction

Due to the development of early detection methods and new treatments including surgery, chemotherapy, radiotherapy, and immunotherapy for cancers, the average survival rates of major cancers are over 50%^1^. However, the five-year survival rate for patients with pancreatic cancer is only 8%^2^. Pancreatic ductal adenocarcinoma (PDAC) is a major histological pancreatic cancer subtype. While surgical treatment offers the only possible cure for PDAC, 80% of patients with PDAC are inoperable at diagnosis. Even after surgery, the five-year survival rate is 15–20%, due to the high PDAC metastatic rate and local recurrence^3^. Currently, chemotherapies or chemoradiotherapies are able to reduce tumor size and improve the prognosis, but these treatments do not fully cure the patients. Morbidity and mortality of PDAC are high in aged people and the world-wide progressive aging of society suggests a rapid increase in pancreatic cancer related deaths in the near future^4^.

Recent studies have shown that cancer stem cells (CSCs) contribute to the heterogeneity of various cancers. The “stem cell theory” of cancer implies that CSCs are responsible for tumor initiation, growth, and metastasis. CSCs are also resistant to chemotherapy and radiotherapy and are believed responsible for tumor recurrence after completion of adjuvant therapy. To examine the heterogeneity of PDAC in detail is one of the major steps required for the development of individualized early diagnosis methods and therapy for patients with PDAC. In addition, various types of cancer cells alter their characteristics from epithelial to mesenchymal (epithelial-mesenchymal transition: EMT) or mesenchymal to epithelial features (mesenchymal-epithelial transition: MET) during growth and the metastatic process^5–8^. We previously reported that the EMT and one of its major regulatory proteins, epithelial splicing regulatory protein 1, correlate to PDAC metastasis^9,10^. Furthermore, the mesenchymal phenotypes of fibroblast growth factor receptors (FGFRs), FGFR-1 IIIc and FGFR-2 IIIc, play important roles in the malignant behaviors of PDAC cells^9,11^.

The characteristics of various cancer cells, including their resistance to anti-cancer drugs, has traditionally been examined using two-dimensional (2D) culture methods. However, the morphology, cell-to-cell and cell-to-matrix adhesions, and cellular differentiation of cells grown in 2D culture systems might differ from those growing *in vivo*^12^. Three-dimensional (3D) cell culture systems are expected to mimic *in vivo* environments^13,14^. To date, many of 3D culture systems have been reported. These include spheroid culture using an ultra-low attachment plate, 3D matrix scaffolds and microsquare patterns on the bottom of the plates, *ex vivo* culture, multilayered postconfluent cell culture, and growing of the cells in extracellular matrix gel^15–17^. Among these 3D culture systems, CSCs are considered to be enriched in the spheres using ultra-low attachment plates^18^, because CSCs self-renew and differentiate in the floating conditions.

In this study, we compared PDAC cells showing epithelial or mesenchymal characteristics in 2D and 3D cultures. Specifically, we assessed cell morphology and the expression and localization of several marker proteins including normal ductal, acinar, tumor proliferation, and TGF-β. Here, we report that 3D culture enhances the morphological and functional differences of PDAC cells showing epithelial or mesenchymal features, and that the epithelial phenotype may be partly maintained due to the ineffectiveness of TGF-β1 induction of the EMT pathway.

## Results

### Quantitative reverse transcription-polymerase chain reaction (qRT-PCR) analysis of EMT marker expression in PDAC cells

**Figure 1:**
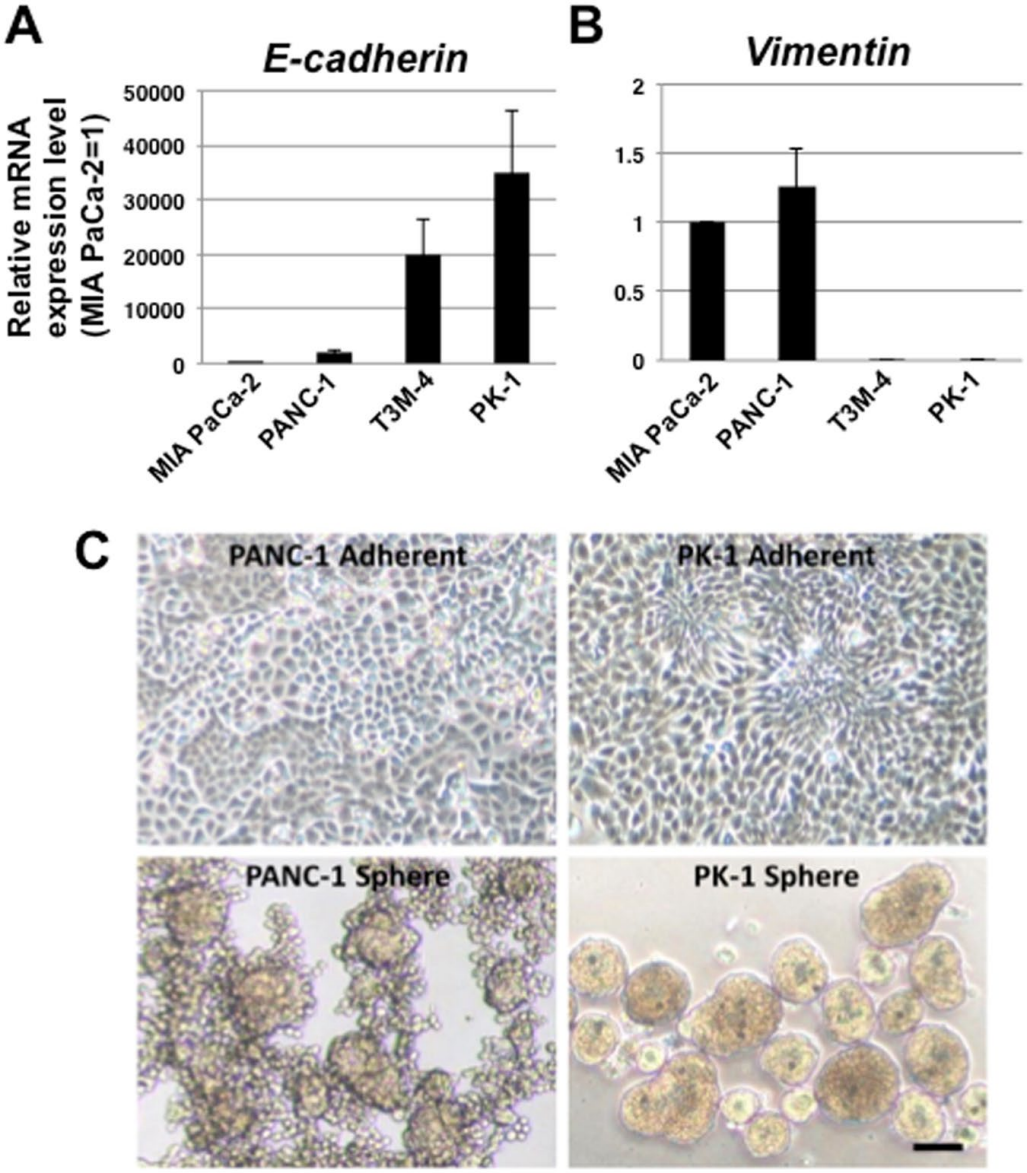
qRT-PCR analysis of four human PDAC cell lines and phase contrast images of PANC-1 and PK-1 under 2D- and 3D-culture conditions. *E-cadherin* and *vimentin* expression levels were examined using qRT-PCR. There was a 35,000-fold difference in *E-cadherin* mRNA levels among the cancer cell lines. (**A**) *Vimentin* and *E-cadherin* expression levels were inversely correlated in each PDAC cell line. (**B**) Results are presented as means ± SD from three independent experiments. The results are shown after normalization to the values obtained for MIA PaCa-2 cells (value = 1). In 2D-culture, PANC-1 cells showed sheet-like proliferation, and PK-1 cells showed a rosette-like appearance (C, upper panel). In 3D-culture, PANC-1 cells formed spheres with a grape-like appearance (lower panel). PK-1 cells formed round to oval-shaped spheres. Scale bar = 200 μm.

To clarify the EMT features of PDAC cells, we examined mRNA expression levels of typical EMT markers, *E-cadherin* and *vimentin*, in four PDAC cell lines. MIA PaCa-2 and PANC-1 cells expressed low *E-cadherin* and high *vimentin* mRNA levels, while T3M-4 and PK-1 cells expressed high *E-cadherin* and low *vimentin* mRNA levels (Fig. 1). *E-cadherin* expression was lowest in MIA PaCa-2 cells and highest in PK-1 cells (Fig. 1A). The *E-cadherin* expression level was 35,000-fold higher in PK-1 cells than in MIA PaCa-2 cells. In contrast, MIA PaCa-2 showed high *vimentin* mRNA levels, but PK-1 cells showed low *vimentin* mRNA levels (Fig. 1B). These results suggest that MIA PaCa-2 and PANC-1 cells have mesenchymal phenotypes, while T3M-4 and PK-1 cells have epithelial phenotypes. PK-1 cells, with the highest *E-cadherin* expression, and PANC-1 cells, with the highest *vimentin* expression, were used for the following experiments.

### Phase-contrast images of PANC-1 and PK-1 cells

When cultured using 2D-culture methods, PANC-1 and PK-1 cells both have pleomorphic cellular morphology. Rosette-like appearances with spindle-shaped cells were observed in PK-1 cells, but not in PANC-1 cells (Fig. 1C, upper panel). When cultured using 3D-culture methods with ultra-low attachment plates, PDAC cells form floating colonies, named spheres^19^. PANC-1 cells formed spheres with cores at the center and a small number of cells localized to the periphery of spheres in a radial pattern. In contrast, PK-1 formed round to oval-shaped spheres with the sphere edge clearly encircled (Fig. 1C, lower panel). These results suggest that there are heterogeneities among PDAC cell lines.

### Immunocytochemical analysis of PDAC cells showing mesenchymal or epithelial features

**Figure 2:**
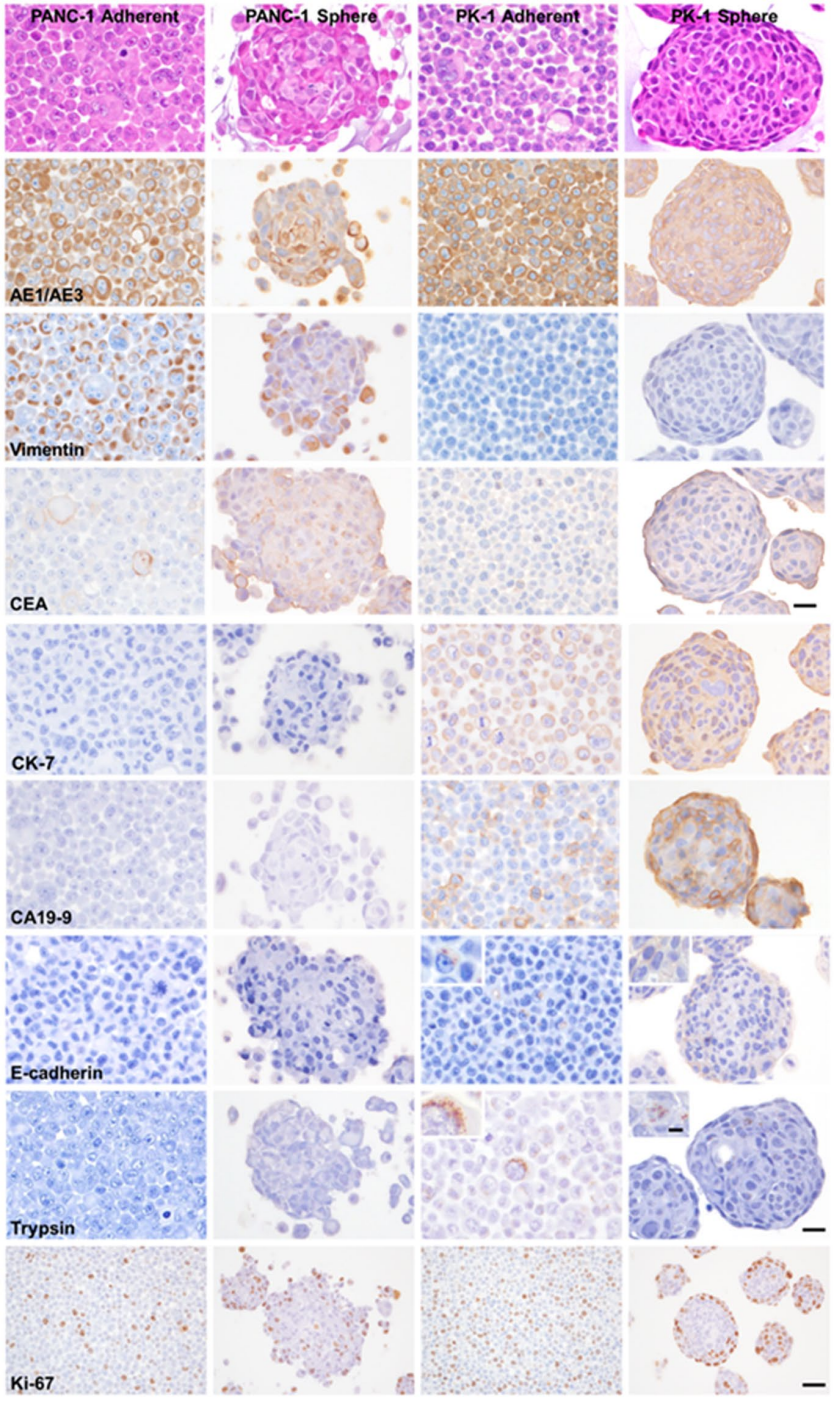
Hematoxylin and eosin staining and immunocytochemical analyses of PDAC cells cultured under adherent and sphere forming conditions. PANC-1 and PK-1 cells grown in the 2D-culture system showed atypical and unevenly localized nuclei and clear large nucleoli. PANC-1 showed round to oval-shaped spheres with a small number of cancer cells at the periphery. PK-1 formed round to oval-shaped spheres, encircled with flat cells. Immunohistochemical analyses of AE1/AE3, vimentin, CEA, CK-7, CA19-9, E-cadherin, trypsin, and Ki-67 in adherent cells and spheres from PANC-1 and PK-1 cell culture. Scale bar = 20 μm, Ki-67: Scale bar = 10 μm, inset: Scale bar = 5 μm.

To clarify the heterogeneities among PDAC cell lines, H&E and immunocytochemical staining were performed using cell blocks from PANC-1 and PK-1 cells cultured under 2D or 3D conditions (Table 1). H&E staining of PANC-1 and PK-1 cells from 2D-cultures showed atypical and unevenly localized nuclei and clear large nucleoli in the cells (Fig. 2). PANC-1 spheres were round to oval-shaped with a small number of cancer cells at the periphery of spheres radially. PK-1 formed round to oval-shaped spheres, encircled with flat-shaped cancer cells. In 2D and 3D cultures, AE1/AE3 (epithelial marker) and carcinoembryonic antigen (CEA, tumor marker) were localized in both PANC-1 and PK-1 cells at variable levels. Vimentin was localized in most PANC-1 cells in both the adherent cells and spheres, and in a small number of adherent PK-1 cells. Vimentin was not detected in PK-1 cell spheres. Cytokeratin 7 (CK7, normal pancreatic ductal marker), CA19-9 (tumor marker), E-cadherin and trypsin (normal pancreatic acinar cell marker) were localized in adherent PK-1 cells and spheres, but not in adherent PANC-1 cells and spheres. These PK-1 cell staining patterns suggest that PK-1 cells have an epithelial phenotype and differentiate to pancreatic ductal or acinar cells. Cell growth patterns were examined using Ki-67 (proliferation marker) immunocytochemical analysis. PANC-1 adherent cells and spheres and adherent PK-1 cells were diffusely positive for Ki-67, but Ki-67 positive cells were localized to the peripheral cells of the PK-1 spheres.

**Table 1:** Summary of immunocytochemistry.

Antibody	PANC-1		PK-1	
	Adherent	Sphere	Adherent	Sphere
AE1/AE3	+++	+++	+++	+++
Vimentin	+++	+++	+	−
CEA	+	+++	++	+++
CK7	−	−	+++	+++
CA19-9	−	−	++	+++
E-cadherin	−	−	+	++
Trypsin	−	−	++	+

Cut off: − (0%); + (<10%); ++ (10–50%); +++ (>50%).

### Scanning and transmission electron microscopy analyses of PANC-1 and PK-1 spheres

**Figure 3:**
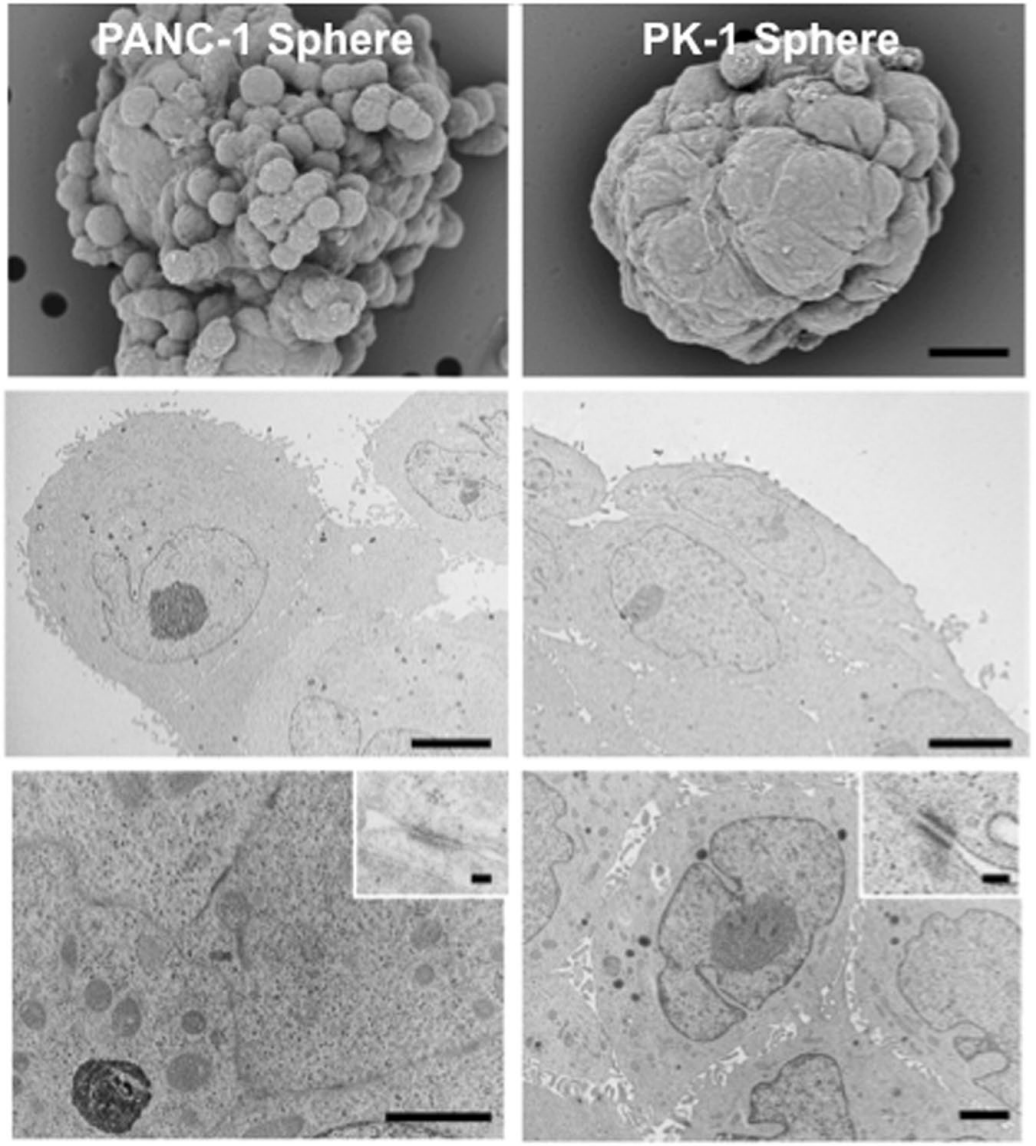
Scanning electron microscopy (SEM) and transmission electron microscopy (TEM) analyses of PANC-1 and PK-1 spheres. SEM analysis showed that PANC-1 spheres were grape-like in appearance and that some cells had protrusions on their surfaces. SEM analysis of PK-1 spheres showed that they were round-to-oval with smooth cell surfaces. TEM analysis revealed the rough cell surface of PANC-1 cells and the lining cells of PK-1 cell spheres. Prominent adherent junctions and desmosomes were observed in PK-1 cells. Microvilli and secretory granules were observed in PK-1 cells. Scale bar: SEM = 10 μm, TEM middle and lower panels = 5 μm, TEM inset = 100 nm.

To examine detailed structural differences between PANC-1 and PK-1 spheres, we performed scanning electron microscopy analysis. PANC-1 formed spheres with a grape-like appearance and sphere-forming cancer cells included cells of varying sizes and surfaces, including protrusions (Fig. 3, left upper panel). PK-1 cells formed round to oval-shaped spheres with smooth cell surfaces (Fig. 3, right upper panel). Transmission electron microscopy revealed some morphological differences between PANC-1 and PK-1 cells in the inner and outer sphere regions. In the outer regions of PANC-1 spheres, cancer cells were separated by intercellular space and were interdigitated with adjacent cells by more microvilli, accompanied by some junctional complex (Fig. 3, left middle panel). The surface of spheres was covered by a layer of flat PK-1 cells with a reduced number of microvilli (Fig. 3, right middle panel). PANC-1 cells of the inner regions had smooth surface and a small number of microvilli and were tightly attached to each other by cell membranes with a few junctional complexes (Fig. 3, left lower panel, inset). PK-1 cells exhibited secretary granules, well developed microvilli, and were connected to each other by marked desmosomes (Fig. 3, right lower panel, inset). In PK-1 cells, the average numbers of desmosomes and adherens junctions at 5,000-fold magnification were 1.4 (25/18 cells) and 0.3 (6/18 cells), respectively. In PANC-1 cells, the average numbers of desmosomes and adherens junctions at 5,000-fold magnification were 0.0 (0/20 cells) and 0.8 (16/20 cells), respectively. The number of desmosomes was significantly higher in PK-1 cells than in PANC-1 cells. MIA PaCa-2 cells showing mesenchymal phenotypes formed grape-like spheres with round- to oval-shaped cancer cells (Fig. S1). No lining cells were observed, and a small number of desmosomes were present in MIA PaCa-2 cells. In addition the morphological characteristics of these cells were similar to those of PANC-1 cells.

### EMT induction in PANC-1 and PK-1 cells

**Figure 4:**
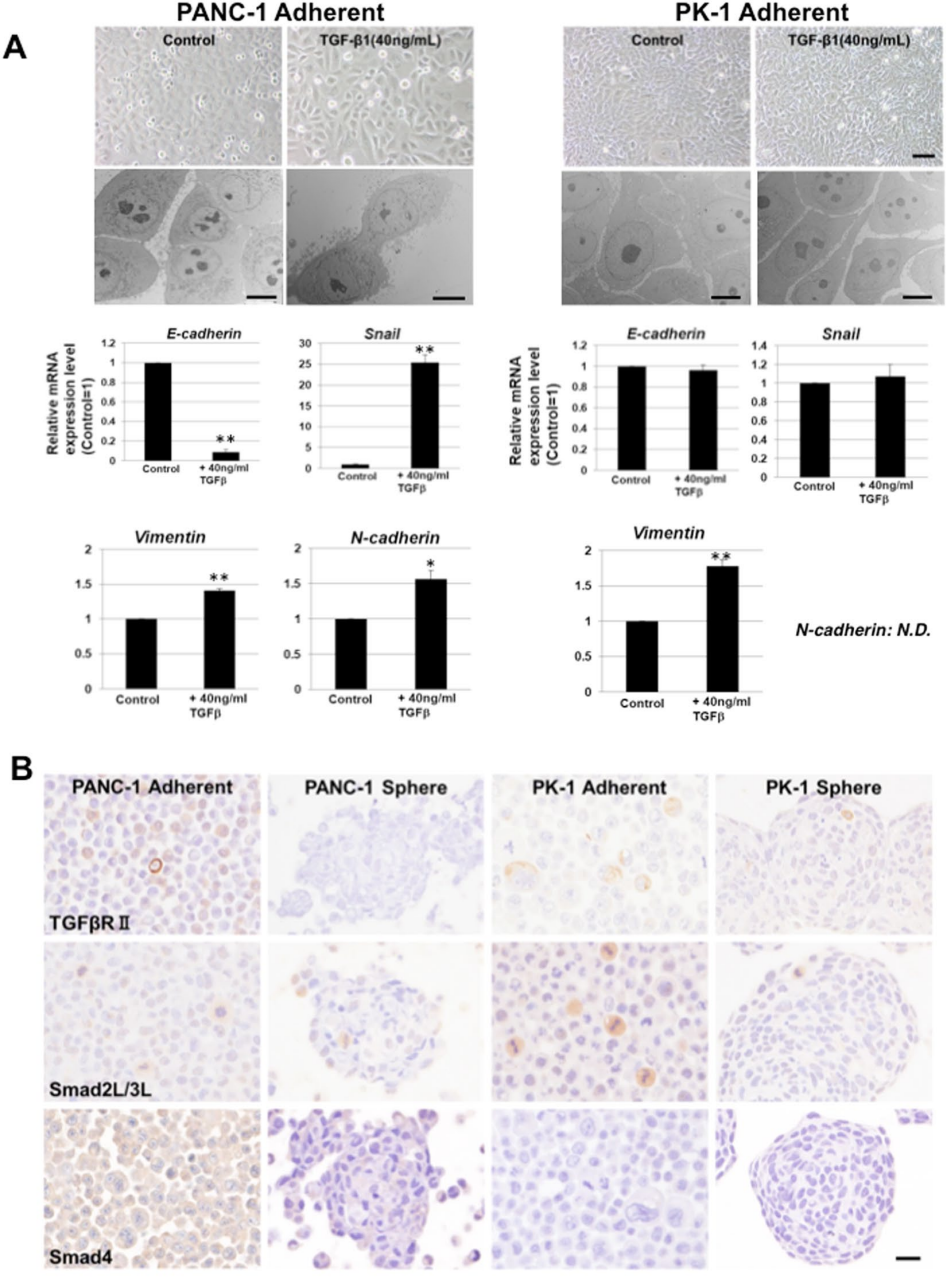
Induction of the epithelial to mesenchymal transition (EMT). (**A**) After TGF-β1 addition, PANC-1 cell morphology changed from polygonal to spindle-shaped, but there no changes were observed in PK-1 cells. TEM analysis showed that spindle-shaped PANC-1 cells had lamellipodia around the cell surface after TGF-β1 administration, whereas PK-1 cells showed no characteristic changes. Expression of *E-cadherin* decreased and expression of *snail* increased in PANC-1 cells. Expression of *E-cadherin* and *snail* did not change in PK-1 cells. Results are presented as means ± SD from three independent experiments. *P < 0.05, **P < 0.01. (**B**) TGFβ receptor II and smad 2 L/3 L were detected in PANC-1 and PK-1 cells, but smad4 expression was not detected in PK-1 cells. Scale bar = 20 μm.

TGF-β1 is a major inducer of the EMT in PDAC cells^5,10,20^. After the addition of TGF-β1, PANC-1 cells altered their morphology from polygonal to spindle-shaped (Fig. 4A). TEM analysis showed a decrease in microvilli and an increase in lamellipodia in PANC-1 cells, whereas no remarkable alterations were observed in PK-1 cells after TGF-β1 treatment. Furthermore, in PANC-1 cells, *E-cadherin* expression was markedly reduced, and *snail*, *vimentin* and *N-cadherin* expression was significantly increased. In contrast, the morphology and expression levels of *E-cadherin, N-cadherin*, and *snail* in PK-1 cells did not change after TGF-β1 administration. *Vimentin* expression was increased in both cell lines. These findings indicate that TGF-β1 induced PANC-1 cells, but not PK-1 cells, to undergo the EMT. Ineffectiveness of the TGF-β1-induced EMT pathway may play an important role in maintaining the PK-1 cell epithelial phenotype.

### Expression of TGF-β1 pathway-related proteins in PANC-1 and PK-1 cells

**Figure 5:**
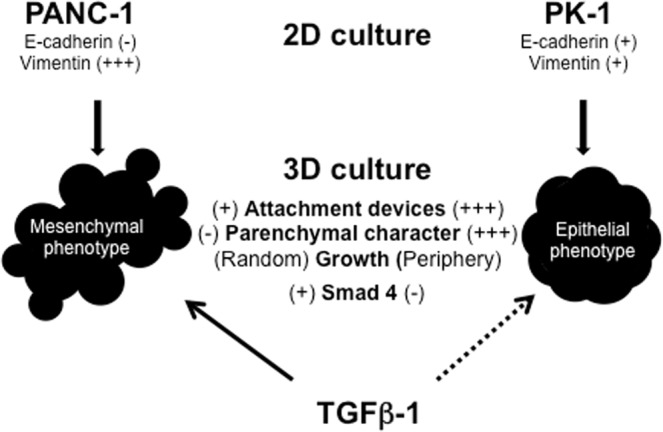
Summary of PANC-1 and PK-1 cell characteristics when cultured under 2D and 3D conditions in this study. Qualitative evaluation was based on immunocytochemical and electron microscopic analyses.

To clarify the mechanisms of the different EMT-responses to TGF-β1, we examined the expression of TGF-β1 pathway-related proteins in PANC-1 and PK-1 cells. Immunocytochemical analysis showed that TGFβ receptor II, which is an initial binding receptor on the cell membrane for TGF-β1, and phosphorylated smad2/3 (smad2L/3L) in the cytoplasm were localized in PANC-1 and PK-1 cells (Fig. 4B). Phosphorylated smad2/3 was strongly expressed in mitotic cells. In contrast, smad4, a TGF-β signaling mediator, was found in PANC-1 cells but not in PK-1 cells. Thus, it is suggested that the ineffectiveness of the TGF-β1-induced EMT pathway in PK-1 cells is partially due to the absence of smad4 (Fig. 5).

## Discussion

In the present study, we identified PDAC cell lines with epithelial or mesenchymal phenotypes based on *E-cadherin* and *vimentin* expression levels. *E-cadherin* mRNA expression levels differed 35,000-fold among the PDAC cell lines. In contrast, *vimentin* expression levels were extremely reduced in the *E-cadherin* expressing PDAC cells. In a previous study, PDAC cell lines were classified into classical and quasi-mesenchymal subtypes based on transcriptional profile analysis^21^. The classical subtype has high expression levels of adhesion-associated and epithelial genes, whereas the quasi-mesenchymal subtype shows high expression levels of mesenchyme-associated genes. PANC-1 and MIA PaCa-2 cells, identified as having the mesenchymal phenotype in our present study, were classified into the quasi-mesenchymal subtype in the published study. These findings suggest that PDAC cells are heterogeneous and that their epithelial or mesenchymal features affect their biological behaviors.

The morphological phenotypes of PANC-1 and PK-1 cells were mesenchymal and epithelial, respectively. These cells had similar malignant pathological features when grown in 2D culture conditions. However, their morphological differences were enhanced when cultured under 3D conditions. PK-1 cells formed spheres with round-like appearance with their peripheries encircled by flat-shaped cancer cells. Surprisingly, Ki-67 immunoreactivity was restricted to the flat cells, indicating that only these cells proliferate in the spheres at same as the proliferating zone in normal epithelial tissues. This proliferation polarity may indicate that PK-1 cells communicate to each other and maintain the characteristics of normal ducts in the spheres. Furthermore, at the center of the PK-1 cell spheres there are differentiated or matured cell-cell attachment devices. PK-1 spheres may expand to outside due to the proliferation of the flat cells and the flat cells may differentiate to cancer cells located inside the spheres. PK-1 cells expressed CK7 and trypsin, normally expressed in pancreatic ducts and produced by pancreatic acinar cells, respectively. These findings suggest that PK-1 also functionally possess epithelial features. In contrast, the Ki-67 proliferation marker was diffusely expressed in PANC-1 spheres and fewer cell-cell attachment devices were observed inside the spheres. The irregular-shaped PANC-1 spheres observed under the 3D culture may arise due to random proliferation. In addition, loss of CK7 and trypsin expression in PANC-1 spheres suggest that PANC-1 cells are both morphologically and functionally undifferentiated. Moreover, expression of vimentin in most PANC-1 cells suggests that the PANC-1 cells have mesenchymal phenotype.

Preparation of cell blocks from the spheres is technically difficult, because spheres are easily broken into their small cellular components. In this study, we avoided centrifugation to collect the spheres and pipetting to mix the cells in formalin. Immunocytochemically, different results were observed for cells grown in 2D and 3D culture systems for CEA, vimentin, CA19-9, E-cadherin, and trypsin staining. Compared to the results observed for cells grown in 2D culture, vimentin expression decreased and E-cadherin expression increased in PK-1 cells grown in 3D culture conditions. More CEA and CA19-9-positive cancer cells were observed in the 3D culture than in the 2D culture. In our recent study, we show that expression levels of the CSC markers *Oct4*, *Nanog*, *Sox2*, *CD24*, and *CD44v9* in PANC-1 and PK-1 cells are higher in spheres than adherent cells^18^. PDAC cells form 3D tumors in the human body, and these findings suggest that 3D culture more clearly represents the physiological functions and characteristics of PDAC cells.

The EMT is induced by the activation of several signaling pathways including TGF-β1, Wnt/β-catenin, and Notch^20,22^. In PDAC, we recently reported that TGF-β1 decreases E-cadherin expression and induces the EMT in PANC-1 cells^17,23^. Here, we found that TGF-β1 did not alter E-cadherin and snail levels in PK-1 cells showing epithelial phenotype. Furthermore, phase-contrast and TEM analyses of TGF-β1-treated PANC-1 cells showed high motility and mesenchymal appearance, including spindle-shaped morphology and lamellipodia formation. Smad4 is a major downstream protein of the TGF- β1 signaling pathway and *Smad4* mutation is one of the four pancreatic cancer cell driver gene mutations^24^. Among TGF-β1 signal related proteins, we could not detect Smad4 in PK-1 cells. Absence of Smad4 in PK-1 cells may contribute to the ineffectiveness of TGF-β1 in inducing the EMT. From this study, we speculate that the inability of TGF-β1 to induce the EMT pathway may maintain the epithelial phenotype in PK-1 cells (Fig. 5). Previous studies have shown that Smad4 expression is correlated to E-cadherin but not vimentin expression after the addition of TGF-β1 in pancreatic cancer^25,26^. Developmental transcription factor Sox4, a transcriptional target of TGF-β1, is able to induce vimentin expression independently of Smad4 in human mammary epithelial cells^26^. Therefore, increased vimentin expression in TGF-β1-stimulated PK-1 cells may be regulated by other intracellular signaling molecules, such as Sox4.

To our knowledge, this is the first report to clarify the morphological and functional differences of PDAC cells with mesenchymal and epithelial features. Further studies using 3D culture are needed to clarify the heterogeneity of PDAC cells containing EMT-features and activation of the EMT pathway, and it will lead to discover early detection methods and treatments for individualized cancer patients.

## Methods

### Cell culture

PANC-1, PK-1, MIA PaCa-2 human PDAC cell lines, were obtained from the Cell Resource Center for Biomedical Research, Institute of Development, Aging and Cancer, Tohoku University (Sendai, Japan). T3M-4 human PDAC cells were provided by the RIKEN BRC through the National Bio-Resource Project of the MEXT, Japan. Cells were grown in growth medium (RPMI 1640 medium containing 10% fetal bovine serum) at 37 °C under a humidified 5% CO_2_ atmosphere.

### qRT-PCR

qRT-PCR was performed as previously reported^17^. Total RNA was isolated from cells using the RNeasy plus mini kit (QIAGEN, Hilden, Germany) and subsequently reverse-transcribed using the ReverTra Ace® qPCR RT Kit (Toyobo, Osaka, Japan). qRT-PCR was performed using the Power Sybr® Green kit (Applied Biosystems, Foster City, CA, USA) and the StepOnePlus™ real-time PCR system (Applied Biosystems). Table 2 lists the primer sets used for qRT-PCR. β-Actin was amplified and used as an internal control. The threshold crossing value was noted for each transcript and normalized to the internal control. The relative quantitation of each mRNA was performed using the comparative Ct method. Gene expression measurements were performed in triplicate.

**Table 2:** List of primer sets for qRT-PCR.

Gene	Forward primer	Reverse primer
*E-cadherin*	CCAGTGAACAACGATGGCATT	TGCTGCTTGGCCTCAAAAT
*Snail*	CCCCAATCGGAAGCCTAACT	GCTGGAAGGTAAACTCTGGATTAGA
*Vimentin*	TCCAAACTTTTCCTCCCTGAAC	GGGTATCAACCAGAGGGAGTGA
*N-cadherin*	TGGGAATCCGACGAATGG	GCAGATCGGACCGGATACTG
β*-actin*	GGTCATCACCATTGGCAATGAG	TACAGGTCTTTGCGGATGTCC

### Sphere-forming assays

To form spheres, cells (1.0 × 10^3^ cells/well) were plated in 24-well ultra-low attachment plates (Corning Inc. Kennebunk, ME) with growth medium as previously reported^27^. After 7 days, the spheres were photographed using a phase contrast microscope (Eclipse TS-100, NIKON). Spheres were then aspirated using micropipettes and used for further experiments. Sphere formation was performed in triplicate in each experiment.

### Cell blocks for adherent cells and spheres

To prepare the cell blocks, adherent cells were collected after trypsin treatment, centrifuged at 1500 rpm for 5 min and fixed with 10% neutral-buffered formalin for 3 h. Spheres were collected using a micropipette under a microscope and fixed in 10% neutral-buffered formalin for 3 h. The formalin was removed using micropipette and cell aggregates were gelled with 1% sodium alginate and 1 M CaCl_2_ and embedded in paraffin. Cell blocks were prepared in triplicate for each cell line.

### Immunocytochemical analysis

Serial sections of the cell blocks (4 μm thickness) were stained with hematoxylin and eosin (H&E) and immunostained using the labeled streptavidin-biotin method. The primary antibodies used in immunocytochemical staining were: mouse monoclonal anti-cytokeratin (CK) AE1/AE3, mouse monoclonal anti-cytokeratin 7 (CK7), mouse monoclonal anti-vimentin, mouse monoclonal anti-CA19-9, rabbit polyclonal anti-carcinoembryonic antigen (CEA) and mouse monoclonal anti-Ki-67 antibodies from Dako (Denmark, Glostrup, Denmark); mouse monoclonal anti-E-cadherin antibody from Takara Bio (Shiga, Japan); mouse monoclonal anti-trypsin antibody from Millipore (Temecula, CA) and mouse monoclonal anti-Smad2L/3L (T220/T179 phosphorylated) antibodies from Immuno-Biological Laboratories (Gunma, Japan); rabbit monoclonal anti-smad4 antibody from Abcam (Cambridge, UK); rabbit polyclonal anti-TGFβ RII (C-16) antibody from Santa Cruz Biotech. (Santa Cruz, CA). Sections were treated with 0.03% H_2_O_2_ in 33% methanol at room temperature for 30 min to block endogenous peroxidase before undergoing antigen retrieval treatment, except for anti-trypsin and anti-TGFβ RII antibodies. The reaction to each antigen was visualized by adding 3,3′-diaminobenzidine tetrahydrochloride chromogen and counterstaining with hematoxylin. Negative control studies were performed by omitting the primary antibody. The immunohistochemical results for the antibodies were evaluated as follows: when the percentage of positive cancer cells was 0%: −, 1–10%: +, 11–50%: ++, 51–100%: +++. Two investigators (Y. S. and K. T.) independently investigated all specimens in a blinded manner.

### Scanning electron microscopic analysis

Spheres from PANC-1 and PK-1 cells were fixed for 2 h with 2.5% glutaraldehyde in 0.1 M phosphate buffer (pH 7.4) at room temperature. Then, the glutaraldehyde solution was removed, and the spheres were washed with PBS. After complete dehydration via a graded ethanol series, sphere samples suspended in 100% ethanol, air-dried, and coated with a platinum layer using an MSP-1S sputter coater (Shinku Device, Ibaraki, Japan). Cells were examined and photographed using a Phenom Pro X desktop scanning electron microscope (Phenom-World BV, Eindhoven, the Netherlands)^27^. Sphere formation was performed in triplicate for observation using SEM.

### Transmission electron microscopic analysis

Spheres from PANC-1 and PK-1 cells were fixed with 2.5% glutaraldehyde in 0.1 M phosphate buffer (pH 7.4), then postfixed for 1 h with 2% OsO_4_ dissolved in distilled water. Cells were then dehydrated using an ethanol gradient, and embedded in Epon. Ultrathin sections were generated using an ultramicrotome and stained with uranyl acetate and lead citrate, as described previously, for examination under a transmission electron microscope (H-7500; Hitachi High-Technologies, Tokyo, Japan)^17,27^. Sphere formation was performed in triplicate for observation using TEM. To observe adherent cells using TEM, we cultured PANC-1 and PK-1 cells on cover slips in 24 well plates. The cells were fixed with 2.5% glutaraldehyde and then post-fixed for 30 min with 1% OsO_4._ After dehydration using graded ethanol, Epon in capsules was placed on the cover slip. Three days later, the cover slip was heated at 100 °C on a hot plate, and then the cells on the cover slip were attached to the Epon. Subsequent procedures were performed as described above.

### EMT induction in PANC-1 and PK-1 cells

For the EMT induction, PANC-1, and PK-1 cells were cultured in growth medium with 20 and 40 ng/ml TGF-β1 for 48 h (PeproTech, Rocky Hill, NJ, USA) as previously reported^5^. EMT induction was performed separately in triplicate for each cell line.

### Statistical analysis

Quantitative data are presented as means ± standard deviations. Differences between two groups were analyzed by Student’s t-test. *P* < 0.05 was considered to indicate a statistically significant difference. Computations were performed using Microsoft Excel 2010 (Microsoft Corporation, Redmond, WA, USA).

## Supplementary information

Supplementary Dataset 1

## References

[1] Allemani C (2015). Global surveillance of cancer survival 1995-2009: analysis of individual data for 25,676,887 patients from 279 population-based registries in 67 countries (CONCORD-2). Lancet.

[2] Siegel RL, Miller KD, Jemal A (2017). Cancer Statistics, 2017. CA Cancer J Clin.

[3] Ahrendt, S. A. & Pitt, H. A. Surgical management of pancreatic cancer. *Oncology (Williston Park)* **16**, 725–734; discussion 734, 736–728, 740, 743 (2002). 12088296

[4] Rahib L (2014). Projecting cancer incidence and deaths to 2030: the unexpected burden of thyroid, liver, and pancreas cancers in the United States. Cancer Res.

[5] Ishiwata T (2016). Cancer stem cells and epithelial-mesenchymal transition: Novel therapeutic targets for cancer. Pathol Int.

[6] Ishiwata T (2018). Role of fibroblast growth factor receptor-2 splicing in normal and cancer cells. Front Biosci (Landmark Ed).

[7] Pang MF (2016). TGF-beta1-induced EMT promotes targeted migration of breast cancer cells through the lymphatic system by the activation of CCR7/CCL21-mediated chemotaxis. Oncogene.

[8] Biddle A (2011). Cancer stem cells in squamous cell carcinoma switch between two distinct phenotypes that are preferentially migratory or proliferative. Cancer Res.

[9] Ishiwata T (2012). Enhanced expression of fibroblast growth factor receptor 2 IIIc promotes human pancreatic cancer cell proliferation. Am J Pathol.

[10] Ueda J (2014). Epithelial splicing regulatory protein 1 is a favorable prognostic factor in pancreatic cancer that attenuates pancreatic metastases. Oncogene.

[11] Kornmann M (2002). IIIc isoform of fibroblast growth factor receptor 1 is overexpressed in human pancreatic cancer and enhances tumorigenicity of hamster ductal cells. Gastroenterology.

[12] Nelson CM, Bissell MJ (2006). Of extracellular matrix, scaffolds, and signaling: tissue architecture regulates development, homeostasis, and cancer. Annu Rev Cell Dev Biol.

[13] Yamada KM, Cukierman E (2007). Modeling tissue morphogenesis and cancer in 3D. Cell.

[14] Vincan E, Brabletz T, Faux MC, Ramsay RG (2007). A human three-dimensional cell line model allows the study of dynamic and reversible epithelial-mesenchymal and mesenchymal-epithelial transition that underpins colorectal carcinogenesis. Cells Tissues Organs.

[15] Matsuda Y (2010). Morphological and cytoskeletal changes of pancreatic cancer cells in three-dimensional spheroidal culture. Med Mol Morphol.

[16] Matsuda Y (2011). Morphological and cytoskeletal alterations of nervous system tumor cells with different culturing methods. Int J Oncol.

[17] Sasaki N (2018). Stemness and anti-cancer drug resistance in ATP-binding cassette subfamily G member 2 highly expressed pancreatic cancer is induced in 3D culture conditions. Cancer Sci.

[18] Sasaki N (2019). Fetal bovine serum enlarges the size of human pancreatic cancer spheres accompanied by an increase in the expression of cancer stem cell markers. Biochem Biophys Res Commun.

[19] Ishiwata T (2018). Pancreatic cancer stem cells: features and detection methods. Pathol Oncol Res.

[20] Miyazono K, Katsuno Y, Koinuma D, Ehata S, Morikawa M (2018). Intracellular and extracellular TGF-beta signaling in cancer: some recent topics. Front Med.

[21] Collisson EA (2011). Subtypes of pancreatic ductal adenocarcinoma and their differing responses to therapy. Nat Med.

[22] Wang Y, Shi J, Chai K, Ying X, Zhou BP (2013). The Role of Snail in EMT and Tumorigenesis. Curr Cancer Drug Targets.

[23] Sasaki N (2018). *H19* long non-coding RNA contributes to sphere formation and invasion through regulation of CD24 and integrin expression in pancreatic cancer cells. Oncotarget.

[24] Korc M (2010). Driver mutations: a roadmap for getting close and personal in pancreatic cancer. Cancer Biol Ther.

[25] David CJ (2016). TGF-beta Tumor Suppression through a Lethal EMT. Cell.

[26] Vervoort SJ, Lourenco AR, van Boxtel R, Coffer PJ (2013). SOX4 mediates TGF-beta-induced expression of mesenchymal markers during mammary cell epithelial to mesenchymal transition. PLoS One.

[27] Ishiwata T (2018). Electron microscopic analysis of different cell types in human pancreatic cancer spheres. Oncol Lett.

