# Supplementary material for: Enhanced morphological and functional differences of pancreatic cancer with epithelial or mesenchymal characteristics in 3D culture
Source: Sci Rep. 2019 Jul 26;9:10871. doi: 10.1038/s41598-019-47416-w (PMC6659675; doi:10.1038/s41598-019-47416-w)
Supplement: Supplementary file 1 — Supplementary Dataset 1 [file 41598_2019_47416_MOESM1_ESM.docx]

**Supplementary Material**

**Enhanced morphological and functional differences of pancreatic cancer with epithelial or mesenchymal characteristics in 3D culture**

Yuuki Shichi, Norihiko Sasaki, Masaki Michishita, Fumio Hasegawa, Yoko Matsuda, Tomio Arai, Fujiya Gomi, Junko Aida, Kaiyo Takubo, Masashi Toyoda, Hisashi Yoshimura, Kimimasa Takahashi and Toshiyuki Ishiwata^*^

Division of Aging and Carcinogenesis, Research Team for Geriatric Pathology, Tokyo Metropolitan Institute of Gerontology, Tokyo 173-0015, Japan

*Corresponding author:

E-mail: tishiwat@tmig.or.jp


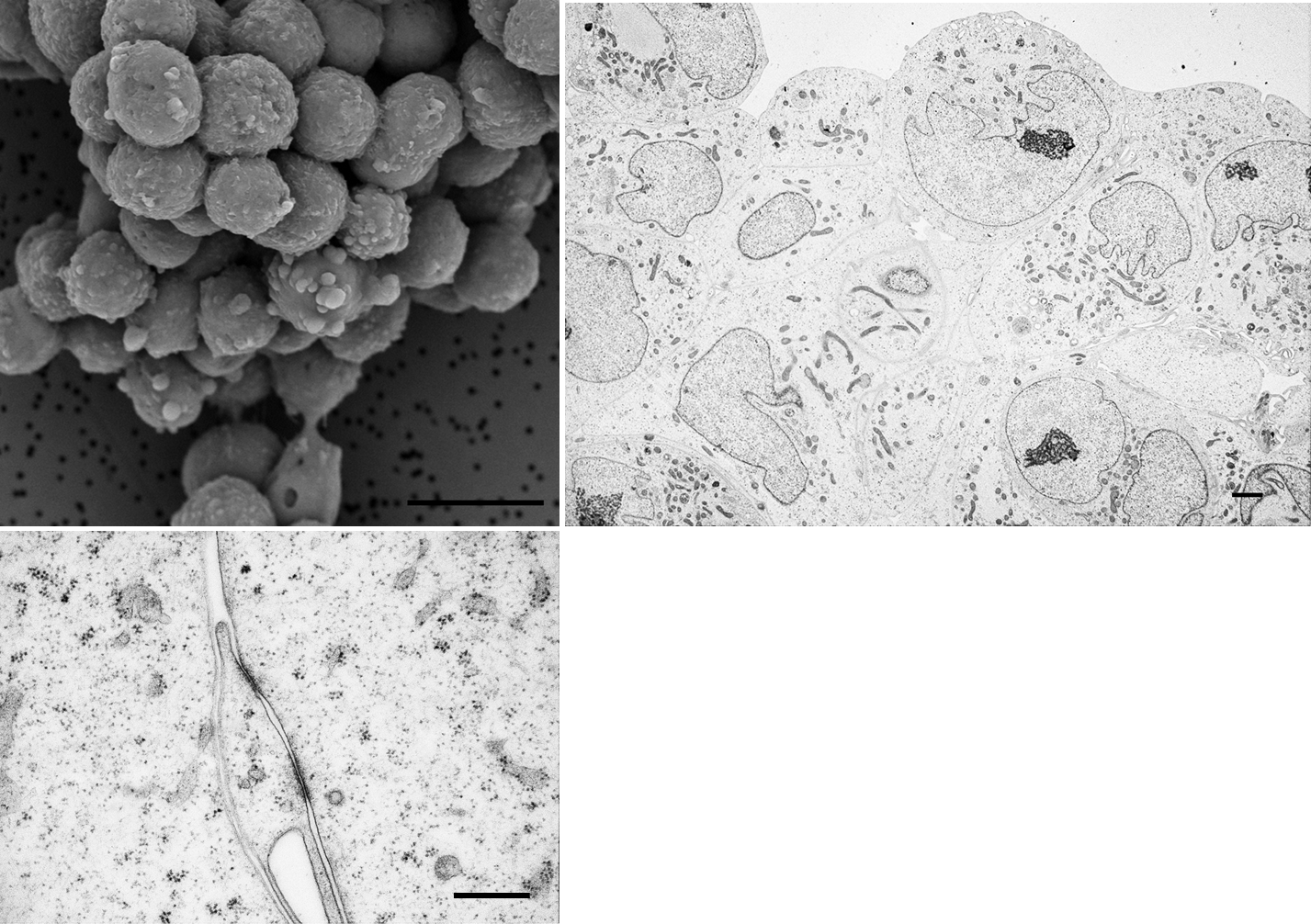


Figure S1. Electron microscopy analyses of MIA PaCa-2 cells. Scanning electron microscopy (SEM) analysis showed MIA PaCa-2 spheres with a grape-like appearance, and transmission electron microscopy (TEM) analysis showed round- to oval-shaped cells with few microvilli (upper right). Desmosomes are observed at cell-to-cell attachments (lower left). Scale bar: SEM = 10 µm, TEM = 2 µm (upper right) and 500 nm (lower left).
